# Health professional student’s volunteering activities during the COVID-19 pandemic: A systematic literature review

**DOI:** 10.3389/fmed.2022.797153

**Published:** 2022-07-19

**Authors:** Tungki Pratama Umar, Muhammad Galang Samudra, Kemas Muhammad Naufal Nashor, Dessy Agustini, Rizma Adlia Syakurah

**Affiliations:** ^1^Medical Profession Program, Faculty of Medicine, Sriwijaya University, Palembang, Indonesia; ^2^Medicine Program, Faculty of Medicine, Sriwijaya University, Palembang, Indonesia; ^3^Department of Health Policy and Administration, Faculty of Public Health, Sriwijaya University, Palembang, Indonesia

**Keywords:** health professional student, volunteer, COVID-19, pandemic, medical student

## Abstract

**Background:**

The Coronavirus Disease 2019 (COVID-19) crisis has forced health and education services to use additional human resources, such as health professional students. Students in the health professions, particularly those in the medical field, can participate in a variety of voluntary activities, both directly and indirectly in health services. The aim of this review was to determine the affecting factors, types of activity, and benefits of undertaking a volunteering role by the health professional student.

**Methods:**

A systematic review of health professional student volunteering during the COVID-19 pandemic was conducted using seven databases: Epistemonikos, ProQuest, Scopus, EBSCOhost, JSTOR, Cochrane Library, and PubMed. This literature search included published articles from March 2020 through to December 2021 using the Preferred Reporting Items for Systematic Reviews and Meta-Analysis (PRISMA) 2020 guidelines.

**Result:**

We included 41 studies that met the selection criteria that assessed the factors and specific programs related to health profession students’ volunteering involvement during the COVID-19 pandemic era. The most frequently observed supporting factor of the eagerness to be a volunteer was the feeling of moral responsibility (such as social dedication, sense of duty, and care), potential learning opportunities, personal interest, and financial compensation. Factors that contributed to a person’s refusal to participate in a volunteer position were the fear of COVID-19 itself (such as transmission, risk of being infected, and personal identification as a risk group).

**Conclusion:**

The review of available literature has shown that understanding the motivation and barriers to the willingness of health professional students to volunteer and the impact of volunteering activities on their future lives is a key for supporting them.

## Introduction

The Coronavirus Disease 2019 (COVID-19) pandemic spread globally, creating a public health and safety crisis. The impact was felt in almost every facet of life, such as health and education services. Due to heavy workloads, health workers were at risk of developing psychological issues such as depression, anxiety, severe stress, and fatigue (1). The COVID-19 pandemic also had an impact on the learning system for students, particularly those in the health professions, by shifting from face-to-face to online learning (2).

In this unprecedented era, e-learning may be an ideal option (3). However, it seems more applicable to preclinical phases of medical education, which are lecture-based. In contrast, clinical stages of medical education oblige students to work in interdisciplinary teams to practice their newly acquired clinical abilities while learning about the healthcare system. Therefore, the shift to e-learning may not facilitate clinical skills and competency acquisition during this stage (3, 4). However, it is understandable that medical schools had to postpone clinical clerkships to reduce student exposure, flatten the curve, and protect healthcare workers during the pandemic due to a lack of personal protective equipment (PPE) (5).

Due to the COVID-19 problem, healthcare services were forced to use additional human resources, such as students from the healthcare professions. This phenomenon was due to the increased pressure on healthcare facilities, caused by the rise of new cases, shortage of doctors, and increased prevalence of burnout among health professionals (6). Students in the health professions, particularly those in the medical field, could participate in a variety of voluntary activities, both directly in health services (triage, admissions wards, hospital clinics, emergency departments, and diagnostic laboratories) and indirectly (call centers, community contact tracing, and community education) (7). The breaks from clinical rotations also provided opportunities for students to engage in academic writing, improve their understanding of critical appraisal abilities, conduct clinical trials, and learn about data analysis (5, 8).

The clinical setting participation varied across different countries and academic institutions. China, Italy, and the United Kingdom integrated medical students into their healthcare systems or graduated them earlier (9, 10). Other countries, such as South Korea and the United States, canceled clerkships to limit patient contact (11, 12). To participate in volunteer work, a student had to be provided with sufficient training, knowledge about their competence, strict supervision, and an adequate supply of PPE (13).

Health professional students volunteered to help the community, profession, and people overcome by COVID-19. From the students’ perspective, this activity was oriented to express loyalty to the health profession, and strengthen a professional sense of belonging (14). However, some students hesitated to volunteer due to various uncertainties, which could cause sleep problems, stress, persistent feelings of discomfort, dread, and anxiety (15). Volunteering offers several educational and social benefits, such as acquiring new skills in real-time data gathering, efficient communication with communities and public health groups, and social media monitoring (16). Therefore, it can help medical students prepare for their future careers as doctors (17). Due to this phenomenon, we conducted a systematic review to determine the motivating factors, barriers, types of activities, and benefits of health professional students volunteering activity during the COVID-19 pandemic.

## Method

### Search strategy

We utilized the Preferred Reporting Items for Systematic Reviews and Meta-Analysis (PRISMA) recommendations for conducting this literature review (18). Before conducting the literature search, the study protocol was approved by all team members. We searched seven databases, including Epistemonikos, ProQuest, Scopus, EBSCOhost, JSTOR, Cochrane Library, and PubMed (Medline *via* PubMed), for published articles from March 2020 through December 2021 that assessed the factors and specific programs related to health profession students’ volunteering involvement during the COVID-19 pandemic era. Hand-searching was also undertaken by examining the references of the selected articles to identify relevant publications that were not indexed in the previously described databases. This step utilized Google Scholar and journals with predominant publications related to health professional education, namely the British Medical Journal (BMJ), BMJ Open, and BMC Medical Education.

We used the following keywords in searching the literature: [(medical student OR health student OR health professional student OR online education OR online teaching OR medical school OR health institute) AND (COVID-19 OR SARS-CoV-2 OR severe acute respiratory syndrome OR nCOV OR coronavirus OR pandemic OR outbreaks OR global crisis) AND (knowledge OR attitude OR practice OR volunteer OR reinforcing factors OR enabling factors OR experience OR opportunity) AND (health education OR health promotion OR health problems OR health support OR hospital OR triage OR tracing OR screening OR monitoring OR disease transmission OR drug administration OR health administration OR disease prevention)]. Two reviewers (TU and DA) searched the databases independently. We did not register our search strategy on the International Prospective Register of Systematic Reviews (PROSPERO) to prevent unwanted delay and allow the data collection process to start immediately.

### Study selection

**FIGURE 1:**
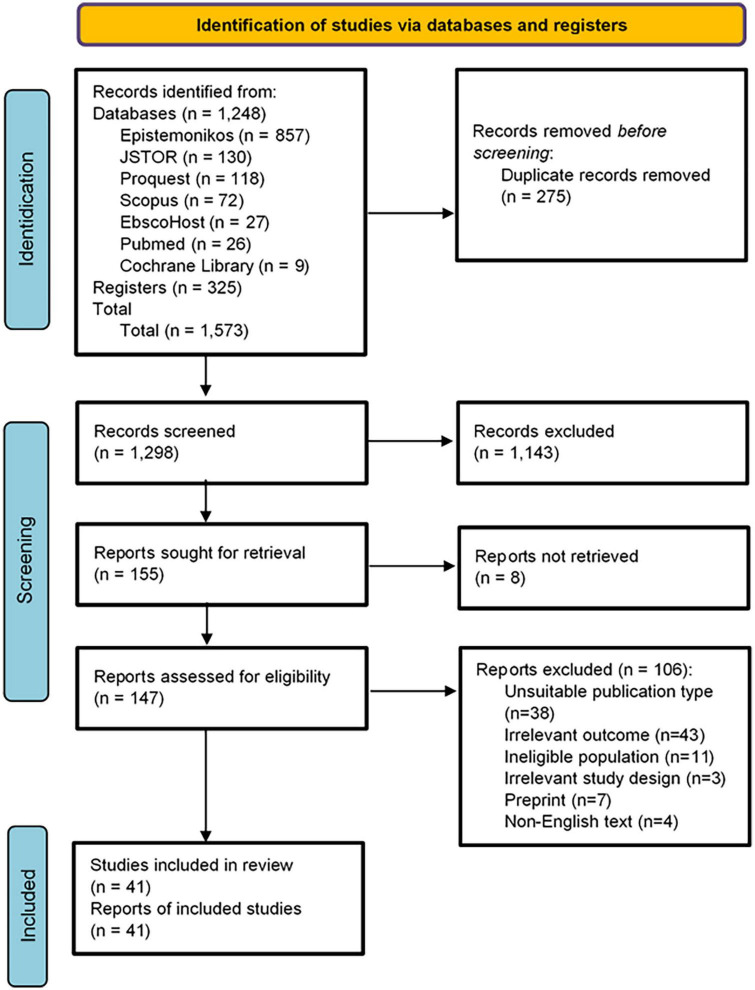
PRISMA 2020 flow diagram of search and data extraction.

We selected qualitative and quantitative (cross-sectional and cohort) studies that assessed the factors and particular programs relevant to the volunteering activity undertaken by health profession students during the COVID-19 pandemic (such as medical, public health, and nursing students). We only used peer-reviewed (excluding the preprints) full-text articles written in English to ensure data accuracy. Articles in the form of literature reviews, case studies, case-control, clinical trials, protocols, conference abstracts, news, editorials, and posters, as well as articles analyzing non-health professional student populations, were excluded. We also decided to exclude studies that explored pooled student populations. Three reviewers (TPU, MGS, and KMNN) independently screened titles and abstracts with semi-automatic processes using Rayyan QCRI, online software for abstract and title screening (19). The duplicates detected by this software were eliminated. Then, the discussion regarding any disagreements related to the title and abstract screening or full-text assessment was undertaken to reach a consensus. From the 1,239 articles obtained, 266 were removed due to duplications. We identified 162 papers for full-text and reference eligibility examination. In the final stage, 41 studies met the inclusion requirements for data synthesis (Figure 1).

### Data extraction and synthesis

Data were extracted and synthesized from the selected studies. Three reviewers (TPU, MGS, and KMNN) extracted relevant information from each study individually, while one author (RAS) with experience in the medical education discipline reviewed the extracted data.

Each article was assessed for quality, such as authorship, years, country, health professional student populations, data collection methods, and key findings. Key findings related to the number of students who were willing to volunteer, motivating factors, benefits of volunteering, obstacles faced, and types of activity were all extracted. Both qualitative and quantitative data were obtained. Risk of bias analysis was determined by using the National Institutes of Health (NIH) Quality Assessment Tool for Observational Cohort and Cross-Sectional Studies checklist (20). The assessment of quality was not used to exclude the studies.

## Result

### Characteristics of included studies

The main characteristics of the included studies are summarized in Supplementary Table 1. The sample size ranged from 12 to 10,433 health professional students (medical and other healthcare professions). Included studies predominantly employed medical students as their study population (31/41; 75.6%), whereas one study also using dental students (21) as their subject population. Seven studies (22–28) (17.1%) included multiple health student disciplines (e.g., public health, nutrition, midwifery, in addition to medical students), while three studies (29–31) (7.3%) exclusively recruited nursing students. Our analysis also covered a wide range of regional distributions, with two studies (32, 33) having a global distribution of respondents. The continents where most of the research originated were Europe, Asia, and America, with 34.1%, 26.8%, and 24.4% distributions, respectively. Meanwhile, Africa had a lesser distribution, with four studies (21, 29, 34, 35) (9.8%) included.

The total number of participants summed from all included studies was 37,000. Fifteen of the 41 studies (36.6%) included students from more than two institutions. The majority of the included studies (85.3%) were cross-sectional in design. Other observed types of study included qualitative and cohort studies, with 12.2% and 2.4%, respectively. Data were mainly taken from the primary source through an online questionnaire or survey (32/41 studies; 78.0%).

### Study quality

**FIGURE 2:**
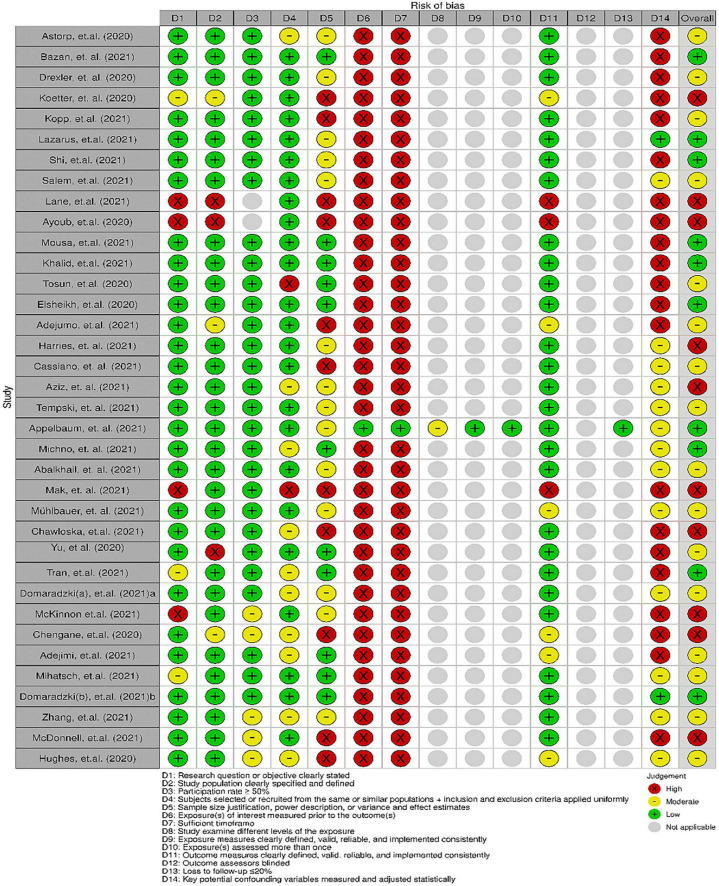
Individual study risk-of-bias assessment using National Institutes of Health Quality Assessment Tool for Observational Cohort and Cross-Sectional Studies checklist.

**FIGURE 3:**
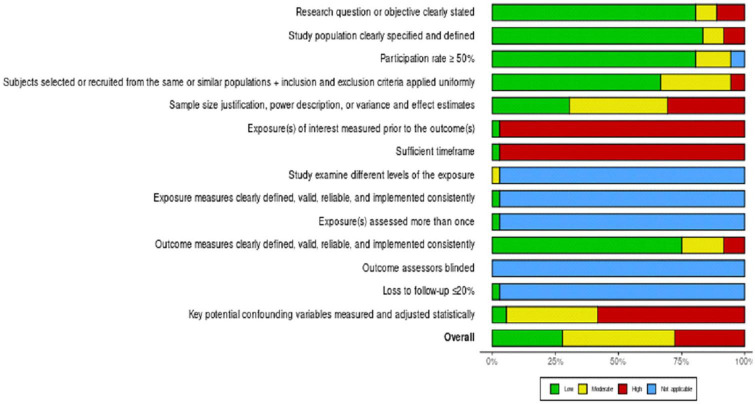
Overall risk of bias on the National Institutes of Health Quality Assessment Tool for Observational Cohort and Cross-Sectional Studies checklist.

We used the National Institutes of Health Quality Assessment Tool for Observational Cohort and Cross-Sectional Studies to assess the likelihood of bias in cross-sectional or cohort studies (Figure 2). Bias in research questions, study demographics, study participation (response rate), recruiting bias, and outcome measures were low (Figure 3). There was a moderate risk of bias since only 26.8% of studies explained the adjustment of their sample size. Meanwhile, a high risk of bias in measuring exposures of interest before outcomes and sufficient timeframes to detect an effect was detected. This phenomenon is mainly related to a large proportion of cross-sectional studies, which only collect data (both exposure and outcome) simultaneously. Only 4.9% of studies measured and accounted for potential confounders, thus mostly having a high risk of bias. Overall, the included studies posed a moderate risk of bias, with an approximately equivalent share between low and high risk of bias studies.

### Factors affecting health profession student willingness of participating as volunteer

Twenty-three papers (56.1%) reported factors impacting health profession students’ desire to volunteer during the COVID-19 pandemic. Seventeen (41.5%) studies focused solely on the medical student population. Meanwhile, six others concentrated on a wider variety of health professional or nursing students. Fourteen studies (34.2%) found a willingness to volunteer ranging from 19.5 to 91.5% (32, 36). However, the actual implementation of this willingness was only observed in a lesser proportion (6.5–67.9%) (25, 37–40), and their readiness level was still low (18.6–58.6%) (32, 38, 41).

The most frequently observed supporting factors for the eagerness to volunteer were: moral responsibility (social dedication, sense of duty, and care), potential learning opportunities, personal interest, provision of adequate PPE, parental support, level of expertise, knowledge, and financial compensation. Lazarus et al. stated that the most significant demographic factors influencing willingness to volunteer were being male, residing in the central part of the country, pursuing education in a public institution, and previous volunteering activity (*p* < 0.05) (41). A chain-mediation analysis of this phenomenon outlined four essential topics: altruistic motivation, prosocial encouragement, self-moral cognition, and reward (42). Included studies showed that in terms of gender, female students (21, 24, 39, 43, 44) were more likely to be involved in the pandemic control and volunteering than males (35, 40, 41), with a willingness proportion of 60.2% (female) vs. 52.3% (male).

Some studies also uncovered elements that led to an individual’s rejection of participating in a volunteer position. The critical factor was the threat of SARS-CoV-2 infection (such as transmission, risk of being infected, and personal identification as a risk group). A scarcity of PPE and the unavailability of a definitive treatment were cited as probable causes of this problem (41). Other observable characteristics included the uncertainty of their academic activity (due to a lack of time for reading, studying, and exams) (45, 46), fear about unfulfilling the volunteer task (associated with qualifications insufficiency) (7, 34), and coming from a lower-middle-income family (41). Another study found that personal perceptions of not being needed by the organization or institution and parental rejection may influence students’ unwillingness to participate in a volunteer program (34).

### Types of volunteering activity

Nine studies described the types of volunteering activities carried out by health professional students during the pandemic (7, 8, 22, 25, 27, 39, 46–48). The activities can be broadly divided into nine different categories, defined as hospital works (triage, admission wards, and emergency room) (7, 25, 27, 46), call center and administration (7, 27, 39, 46), epidemiological aspects (contact tracing, testing) (22, 47, 48), online or remote consultation (regarding COVID-19 or non-COVID-19 cases, using phone or internet) (46–49), laboratory-related works (47), food and/or PPE supply (27, 39), mentoring juniors (39), public education (such as countering hoaxes) (48), and in research programs (8). Some students also reported participating in more than one type of volunteering activity (7, 39).

### Benefits of volunteering activity

Fourteen studies revealed the benefits of participating as a volunteer during the current pandemic from the health professional students’ perspectives (7, 8, 16, 17, 22, 24, 27, 28, 46, 47, 50–53). The most frequently mentioned advantage was to learn and practice, especially to give real aid and explore evidence-based medicine (8, 16, 17, 22, 24, 28, 47, 53). Other benefits included collaborating with non-physicians (8, 22, 47), strengthening communication skills and empathy (8, 16, 17, 22, 24, 50, 54), knowing more about the healthcare system and costs (22), developing leadership and time management (24, 47, 52), helping other people (social benefits) (16, 17, 24, 28, 51), getting recognition (from friends, other healthcare workers, patients, etc.) (7, 46), providing an interactive learning platform (49), receiving financial compensation (52), and experiencing a research atmosphere (8). Following the positive impression of volunteering activity, three studies found a high level of willingness (73.2–94%) among health professional students regarding participation in the future (7, 49, 53). Regarding mental issues associated with volunteering, four studies found that student volunteers had low psychological stress (40, 44, 51, 55). The prevalence of anxiety and depression was lower among volunteering students than non-volunteering ones, indicating that it positively influenced general psychological wellbeing (40, 51).

## Discussion

The COVID-19 pandemic has made substantial changes in social life. Due to societal constraints during this pandemic, most of the included studies in this review relied heavily on primary data collected *via* online questionnaires or surveys. This method mainly provides quick, easy, and economical way to obtain large samples (56).

The current COVID-19 pandemic has pushed everyone to contribute. As future healthcare providers, health professional students are regarded as those with the closest capability to assist (57). This review analyzed health professional students’ willingness to volunteer in pandemics, in addition to their motivation, benefits, and obstacles to volunteering. Health professional students’ desire to participate as volunteers are influenced by moral responsibility, personal interest, social dedication, prosocial motivation, self-cognition, and learning opportunities. The majority of health professional students were willing to fight the pandemic (41, 46). The previous studies in Ireland also showed that the majority of health professional students would volunteer during pandemics (58).

There were various reasons for health professional students’ eagerness to volunteer during the COVID-19 era. A shortage of medical personnel and sense of duty were the main reasons supporting the students’ willingness to volunteer (41). Gender, volunteering experience, types of academic institution, place of living, and family income had higher scores for willingness and readiness to volunteer (41, 59). Female students were more likely than male students to volunteer in pandemic control (60.2 vs. 52.3%). This finding is consistent with previous research, which found that women were more inclined to volunteer because of their nurturing, generous, and empathetic nature, but for a shorter period than male participants (60, 61). Moreover, health professional students with high prosocial motivation were more likely to engage in volunteer behavior during a pandemic crisis (42). This is in accordance with the findings from the previous studies that showed that increases of prosocial motivation lead to increases in either work or volunteering behavior (62).

Most of the students indicated that volunteering activities provided direct benefits such as gaining a sense of giving direct aid, building professional experience, and developing collaboration skills (7). This may be due to health-related activities influencing health professional students to contribute (63). These findings are consistent with the previous studies (64–66). Thus, giving motivation to the health professional students to contribute as volunteers in this pandemic era became crucial. Another advantage of volunteering is that it helped prevent mental-health problems during this uncertain time. Volunteering was also linked to improved mental wellbeing (67).

On the other hand, some barriers may be experienced by health professional students discouraging them from participating in volunteer work during COVID-19. Fear for their own health, the lack of a treatment, and the fear of harming patients were key factors limiting their willingness to volunteer (41). Health profession students responded that their safety while working was a priority (45). As a result, the regulation on this topic appears to be necessary as a prerequisite (7). Fostering volunteerism among medical students requires the joint effort of the government, non-profit organizations, hospitals, and medical colleges (42). Government and all-level organizations should contribute to create extensive job opportunities and platforms for medical students to generate volunteer services, as well as to build a sustainable incentive system to encourage medical students to engage in volunteer behavior to serve society (42). Training and education were related to update the safety recommendations (68). Hospitals should give the training sessions and theoretical prequalification before health professional students start activity in volunteering (45). The university should provide clear protocols and guidance for volunteering activities (45). Senior colleagues should further address and support safety during clinical work (45).

Health professional students can undertake many activities to contribute in the response to the COVID-19 pandemic. Nine studies included in this review assessed the form of activities that health professional students undertook to fight the pandemic. The activities can be broadly divided into nine different categories, defined as direct patient care (7, 25, 27, 46), call center and administration (7, 27, 39, 46), epidemiological aspects (22, 47, 48), online consultation (46–49), laboratory-related works (47), equipment supply (27, 39), mentoring juniors (39), health promotion and education (48), and in research programs (8). Some students also reported being involved in more than one type of volunteering activity, such as in patient’s triage and admission wards (7, 39). This is important in increasing public knowledge and awareness, supporting healthcare facilities, and evidence-based practice regarding the COVID-19 pandemic (7, 17, 69).

Our work provides an exclusive systematic review of volunteering activities undertaken by health profession students during the COVID-19 pandemic. The previous research, although also emphasizing volunteerism and readiness in the case of a pandemic, is mainly tracked back to past scenarios, therefore, explaining different pandemic or disaster situations. Furthermore, during the COVID-19 pandemic, we evaluated the determining factors, types of activities, and advantages of volunteering (which was exclusively done in this systematic review). Moreover, this study employed a larger population of health professional students (medical and other healthcare professions) than previous research (only inclusive of medical students) (70).

Although the majority of medical students were willing to voluntarily support the care system during the pandemic, only a small proportion of them had adequate readiness to practice (41). This is in agreement with the findings of previous research in Germany, which revealed relatively low degrees of practice preparedness (58). This means that further preparations are required to ensure that they have sufficient knowledge and skills (57). The experience of volunteer service and the impact on healthcare students’ life were identified (71). They were able to work for a longer period, more hours in addition to displaying a higher level of satisfaction and confidence, and when given adequate encouragement and valued by medical staff (46, 63). Volunteering activity can provide the opportunity to learn and practice skills in collaboration, communication, and health systems’ science (22).

**FIGURE 4:**
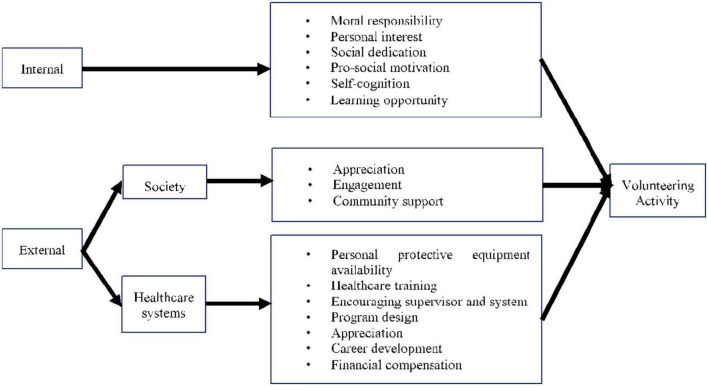
Proposed Model of Volunteering activity in Health Professional Students.

There are some limitations found in this systematic review. The number of samples was highly variable (the smallest was 12, and the largest was more than 10,000). The study with a small sample size may not represent all health professional students. Moreover, many studies had a moderate risk of bias due to their cross-sectional design (attributed to insufficient observation) and lack of confounder adjustment. The heterogeneity of the outcome measures changed the pattern of this review. Due to large geographical distribution, there may be cultural disparities among them, although it is helpful to picture a global phenomenon of volunteering in health professional students. The scope of this review is broad enough because it discusses the motivation and barriers to the willingness of health professional students to volunteer, the types of volunteering activities that health professional students can undertake, and the impact of volunteering on their future lives. We also developed a proposed model of volunteering activity in health professional students to summarize the findings (Figure 4).

## Conclusion

The review findings highlight the affecting factors, types of activity, benefits, and obstacles of undertaking a volunteering role by health professional students during the COVID-19 pandemic. Understanding the motivation and barriers to the willingness of health professional students to volunteer and the impact of volunteering activities on their future lives is a key for supporting them. Additional studies with larger sample sizes, equal sample distribution, and with adjustment of confounders related to COVID-19 volunteering by health professional students are needed.

## Data availability statement

The original contributions presented in this study are included in the article/Supplementary Material, further inquiries can be directed to the corresponding author.

## Author contributions

TPU, DA, MGS, KMNN, and RAS: literature review concept design and literature search. TPU, DA, and RAS: figures and tables. RS: reviewed each extracted data. All authors contributed in the analysis and interpretation of data, drafting of manuscript, and approval of the final manuscript.

## Conflict of interest

The authors declare that the research was conducted in the absence of any commercial or financial relationships that could be construed as a potential conflict of interest.

## Publisher’s note

All claims expressed in this article are solely those of the authors and do not necessarily represent those of their affiliated organizations, or those of the publisher, the editors and the reviewers. Any product that may be evaluated in this article, or claim that may be made by its manufacturer, is not guaranteed or endorsed by the publisher.
