# Supplementary material for: Health professional student’s volunteering activities during the COVID-19 pandemic: A systematic literature review
Source: Front Med (Lausanne). 2022 Jul 19;9:797153. doi: 10.3389/fmed.2022.797153 (PMC9345056; doi:10.3389/fmed.2022.797153)
Supplement: Supplementary file 1 [file Table_1.docx]

Supplementary Material

# Supplementary Data

The original contributions presented in the study are included in the article/ Supplementary Materials, further inquiries can be directed to the corresponding author/s.

# Supplementary Figures and Tables

## Supplementary Tables

**Table 1. Summary and key findings of included studies**

| Author (year) | Country | Study type | Health student populations | Primary aim | Data collection tools | Key findings |
| --- | --- | --- | --- | --- | --- | --- |
| Abalkhail et al. (2021)^59^ | Saudi Arabia | Cross-sectional | 410 undergraduate medical students | Describe the personal and family-related aspects that influence undergraduate students' willingness to volunteer during the pandemic | Online survey questionnaire | - Medical students tend to volunteer (60.7%). - The respondent's living status (either with families, friends, or alone) influenced their likelihood of volunteering. - Volunteerism was not hampered by inadequate training or infected coworkers. |
| Adejimi et al. (2021)^21^ | Nigeria | Cross-sectional | 548 medical/ dental students | Assess the attitudes and perceptions  of medical and dental students about volunteering during the COVID‑19 pandemic | Online survey questionnaire | - The majority of respondents were willing to volunteer if supplied with sufficient PPE (82.9%) and training (79.3%) - Penultimate year students were more inclined to volunteer during human health resources’ shortages. Final-year students were more inclined to volunteer if prompted by the government - Female students were more likely than males to volunteer (OR:2.44) |
| Adejumo et al. (2021)^29^ | Nigeria | Cross-sectional | 726 nursing students | Evaluate Nigerian undergraduate nursing students' knowledge, attitude, and willingness to volunteer for the COVID-19 pandemic. | Online survey questionnaire | - 62.8% of respondents were willing to be volunteers during the COVID-19 pandemic - Financial compensation (money, food, and transport allowances), moral obligations, adequate PPE supply, and training were the main causes for medical students to volunteer |
| Ali et al. (2021)^17^ | Ireland | Qualitative | 26 medical students | Measure students' professional development, resilience, and perceived career choices after volunteering during the COVID-19 pandemic | Online survey | - Communication, teamwork, compassion, altruism, evidence-based practice, and self-care were established by volunteers during the COVID-19 pandemic - Difficulties in volunteering were due to lack of clinical exposure, therefore, the students need additional support |
| Appelbaum et al. (2021)^39^ | United States | Cohort | 518 medical students | Investigate socio-demographic differences in medical students' educational preferences, attitudes, and volunteerism during the COVID-19 pandemic | Online survey questionnaire | - During the COVID-19 pandemic, 55.5% of medical students volunteered - There were 15 volunteer categories, primarily in food/nutrition, mentoring premeds, and call centers. Some students volunteered for multiple activities - More second and third^-^year medical students volunteered than expected. Meanwhile, fourth and first-year medical students volunteered less. Gender or prior experience with disaster was not associated with this phenomenon - First and second-year female students were more inclined to volunteer in nonclinical healthcare settings - Participants agreed more that students could help in nonclinical settings during a pandemic two months after being banned from clinical settings |
| Astorp et al. (2020)^45^ | Denmark | Cross-sectional | 486 medical students | Identify medical students’ motives to contribute as a pandemic emergency medical response team | Survey questionnaire | - The top priority for medical students to keep working is their safety - Before students begin clinical practice, hospitals should discuss this topic during theoretical prequalification and training sessions - Senior colleagues should focus on and promote clinical safety even more - University’s encouragement to join the pandemic emergency medical response teams was mentioned as a motivator by students |
| Ayoub et al. (2020)^47^ | United States | Cross-sectional | 151 medical students | Describe the involvement of Ochsner Clinical School medical students as volunteers in the COVID-19 pandemic | Direct observation | - The highest proportion (71%) of student volunteers was in the third and fourth year - Medical student volunteers can undertake some roles, including the COVID-19 hotline, laboratory, telemedicine, family communication, and COVID-19 diagnostic testing - The voluntary role may benefit students, including increased knowledge regarding medicine, the importance of nonphysician roles in healthcare teams, and empathy and listening skills - Some opportunities may arise for the students, including serving their community, gaining new experiences to practice their clinical skills, and acquiring and developing valuable leadership skills |
| Aziz et al. (2021)^30^ | Brunei Darussalam | Cross-sectional | 72 nursing students | Investigating Bruneian nursing students' willingness to volunteer during a pandemic | Online survey questionnaire | - 75% of individuals expressed a willingness to volunteer during the COVID-19 pandemic - Marital status (married, p < 0.001), personal and family safety (p < 0.001), year of study (junior, p < 0.001), altruism (p < 0.001), and knowledge level (p < 0.001) significantly influenced the willingness to volunteer - The most significant barrier to nursing students volunteering during the COVID-19 pandemic was caring for dependent children (53.6%). Other impediments included a lack of crisis preparedness knowledge and personal safety concerns |
| Bazan et al. (2021)^7^ | Poland | Cross-sectional | 580 medical students | - Describe the general characteristics of volunteers - Describe the scope of their efforts in combating COVID-19 - Explore the fears and obstacles throughout volunteering activity - Assess the level of satisfaction and benefits arising from undertaking voluntary work | Online survey, structured questionnaire | - Medical students were the greatest contributor of volunteers - The most frequent tasks included patient’s triage (> 55%), operating patient call centers, and helping medical professionals in admission wards (> 25%) - Most students indicated that volunteering activities provided direct benefits, mostly related to the sense of giving real service, professional experience, and collaboration skills - It is critical to ensure medical students’ safety and protection at healthcare units while volunteering during the COVID-19 pandemic - Longer volunteering periods and working hours were related to a higher level of satisfaction from volunteering |
| Cassiano et al. (2021)^38^ | Brazil | Cross-sectional | 125 medical students | Identify the prevalence of volunteering students during the COVID-19 pandemic and the factors that affected it | Online survey questionnaire | - 52% of students volunteered, with seven doing community service, eight doing medical night calls, 16 with Expeditionary Health, and 56 with telemedicine - The majority of the volunteers were fifth or sixth-year medical students (66.15%). Meanwhile, the fraction of fourth-year medical students was 33.8%. However, in a specific view, only 45.7% of fifth and sixth medical students volunteered, compared to 71.0% of the fourth-year medical students - 77.6% of students felt incompetent, and 78.4% felt inadequate in coping with a global health emergency - More time spent on social media lowered volunteerism - Students from high-income families (receiving 5.1–10 times the minimum standard wage), fourth-year medical students, and students who felt more confident and capable of coping with the pandemic were more inclined to volunteer |
| Chawloska et al. (2021)^16^ | Poland | Cross-sectional | 158 medical students | Identify student volunteers’ perceptions on the situations, safety, costs and beneﬁts of their involvement in the volunteering projects | Online survey questionnaire | - Student volunteering could help health professional’s education. Volunteers’ needs must be examined, psychological assistance supplied, and opportunities for mentoring and reflection provided for optimal implementation - Most respondents said they learned new and fascinating things. Most noted an improvement in soft skills (social, organizational, stress management, and medical skills). Other benefits for students included learning about healthcare systems and costs |
| Chengane et al. (2020)^28^ | United States | Cross-sectional | 33 health students | Describe Student Response Team (SRT) and make a foundation, training, and development through academic institutions across the world, during and beyond the COVID-19 pandemic | A training is planned for each semester to prepare volunteers | - Volunteering with the SRT appealed to students for four reasons: community service, pandemic experience, training, and professional exposure |
| Domaradzki et al. (2021)^26^ | Poland | Cross-sectional | 417 healthcare students | Determine the relationship between religion and the motivations of future healthcare professionals to volunteer during the COVID-19 pandemic | Online questionnaire | - While religiosity was not a strong predictor of volunteering during the pandemic, it did play a role in students’ motivations to join the fight against COVID-19 and their desire to serve the community |
| Domaradzki et al. (2021)^27^ | Poland | Cross-sectional | 417 healthcare students | Describe students’ experience of the pandemic as well as their experiences with a volunteer service during the COVID-19 pandemic | Online survey questionnaire | - Students were primarily involved in administrative work (39.8%), emergency room assistance (33.3%), taking patients’ medical histories (21.3%), assisting with medical procedures in a hospital ward (18%), making supplies of PPE, and providing telephone advice in a sanitary-epidemiological station (7%) - The motivations for volunteering were the moral view that medics should participate and help, the importance to aid others, a desire to be a part of something significant, and a desire to gain experience for a future profession - Reasons for volunteering included helping others, giving something to the community, and building their resume |
| Drexler et al. (2020)^46^ | Germany | Cross-sectional | 137 medical students | Evaluate the working fields of volunteer students and the impact of the pandemic on final-year students from a student’s standpoint | Survey on institutional online data collection program | - The majority of medical students were willing to make a significant contribution in the response to COVID-19 (70.1%) - The majority of students thought that their effort was valuable and that medical professionals valued it, especially in their volunteer capacity - Students who volunteered assisted doctors with patient care or worked at the COVID-19 hotline service - The most significant factor affecting their willingness to join the volunteering activity was a form of social commitment and sense of duty of future healthcare practitioners - 73.2% of students would like to be involved in the voluntary activity again |
| Elsheikh et al. (2020)^34^ | Sudan | Cross-sectional | 622 medical students | Identify and determine the knowledge and willingness of medical students to participate in response to the COVID-19 pandemic in Sudan | Online survey questionnaire | - 57.2% of Sudanese medical students wanted to be volunteers during the COVID-19 pandemic - Unwillingness of the medical students was due to lack of qualifications, personal perception of not being needed by the organization, parental rejection, and fear of SARS-CoV-2 infection - Only residence state was associated with willingness to help restrict COVID-19 among Sudanese medical students (p = 0.012) |
| Findyartini et al. (2020)^48^ | Indonesia | Qualitative study | 250 undergraduate medical students | Examine medical students’ adaptation during a pandemic via the viewpoint of Professional Identity Formation | Students’ written reflections | - Preclinical and clinical students can volunteer to help educate the public about COVID-19, stop the spread of hoaxes, raise funds for frontline workers’ PPE and nutrition, do contact tracing, and monitor asymptomatic patients during self-isolation |
| Harries et al. (2021)^36^ | United States | Cross-sectional | 741 medical students | Investigate the psychological and educational implications of the COVID-19 pandemic on US medical students and their reactions to the Association of American Medical Colleges (AAMC) proposition | Survey questionnaire | - In non-shortage healthcare worker conditions, 63.5% of medical students consented to volunteer, but that dropped to 19.5% in shortage circumstances - 37.8% of medical students agreed that they had a moral, ethical, or professional obligation to contribute during the pandemic |
| Hughes et al. (2020)^50^ | United Kingdom | Cross-sectional | 33 medical students | Assist patients who are at high risk of contracting COVID-19. The project would bridge the digital divide, enabling proactive, anticipatory preparation and creating an active learning environment to compensate for the pandemic’s influence on medical education | Quantitative process and descriptive data and individual qualitative interviews | - Respondents felt students brought significant practical and psychological benefits, especially in increasing community volunteer groups’ relationships - Students enjoyed the project and appreciated the vibrant learning atmosphere and reflective feedback sessions - This study shows how medical students can help vulnerable patients while fostering active medical education |
| Khalid et al. (2021)^37^ | Pakistan | Cross-sectional | 200 medical students | Assess medical students' willingness, motivations, and challenges to volunteer work during a pandemic | Online survey questionnaire | - Most medical students (46%) intended to assist during the pandemic, but only 6.5% did - Second-year medical students and those attending their preferred hospital (COVID-19 or non-COVID-19) were more inclined to volunteer - Sufficient PPE, parental support, adequate expertise, treatment coverage, separate accommodation, and psychological support were all reinforcing factors for the medical students willing to undertake voluntary activity - The medical student’s willingness to volunteer was not influenced by moral obligation or official sanction |
| Koetter et al. (2020)^22^ | United States | Cross-sectional | 110 health professional students | Disseminate the contact tracing procedure, including phone scripts and workflow, to other health systems for use in the COVID-19 pandemic and future public health crisis response efforts | Health Insurance Portability and Accountability Act (HIPAA) compliant Research Electronic Data Capture (REDCap) | - Health professional students were allocated to trace cases and their contacts to suppress the pandemic by using phone calls or e-mail - Volunteering may give students the chance to acquire and practice collaboration, communication, and health system science skills |
| Kopp et al. (2021)^49^ | United States | Cross-sectional | 12 medical students | Describe the development and implementation of remote eConsult program as well as the experiences of student and faculty participants. | Survey on inpatient eConsult program | - The eConsult program was used for assessing consultation regarding COVID-19 and internal disease problems (topics on nephrology and secondary infection) - Nine (75%) students were willing to be volunteers again |
| Lane et al. (2021)^23^ | United States | Cross-sectional | 423 health professional  students | Describe the deployment of medical students to provide emergency childcare throughout the COVID-19 pandemic | Online survey questionnaire, Twitter | - Student volunteers may be involved in emergency childcare services by applying clear communication, grassroots organizing, patient triaging, and leveraging new technology |
| Lazarus et al. (2021)^41^ | Indonesia | Cross-sectional | 4,870 undergraduate medical students | Evaluate the willingness of Indonesian medical students’  to volunteer and their readiness to practice during the COVID-19 pandemic | Online self-reported questionnaire | - While 48.7% of the students expressed their willingness to participate in volunteering activity, only 906 (18.6%) were adequately prepared to practice - The most significant factors affecting willingness to volunteer were being male, prior volunteering experience, students from public universities, and living in Central Indonesia - The most significant factors affecting readiness to volunteer were being male, prior volunteering activity, living in Central Indonesia or Sumatra, age, undergoing clinical phase of education, and from public university - The students’ willingness to volunteer was boosted by a sense of duty, a shortage of medical personnel, and requests from stakeholders - Fear for their own health, the lack of a treatment, and the fear of harming patients, on the other hand, were the key factors limiting their willingness to volunteer |
| Mak et al. (2021)^53^ | United Kingdom | Cross-sectional | 235 medical students | Perform a retrospective computerized survey of second-and third-year Warwick Medical School students who volunteered during COVID-19's first peak | Online survey questionnaire | - Medical students found volunteering during the COVID-19 pandemic a positive experience, enabling them to acquire skills that would likely be useful for the rest of their training - 94% of students would like to volunteer again if needed |
| McDonnell et al. (2021)^54^ | United States | Cross-sectional | 71 medical students | Examine student motivations to provide insight into how medical schools might engage student volunteers with the elderly during and after the COVID-19 pandemic | Online survey questionnaire | - The main reasons for involvement were classified as “value-based” (62%), which included compassion toward their isolation and concerns for the older population, “understanding” (17%), with many students wanting to learn about older adults through practicing communication skills, “enhancement” and “protective” aspects of the self (17%), and gain experience (4%). - No one reported “social” motivators |
| McKinnon et al. (2021)^8^ | United Kingdom | Cross-sectional | 224 medical students | Gain insights about medical students’ perceptions of Volunteering in Research program. | Online survey | - Students reported learning about clinical trials and documentation, COVID-19 disease and therapies, interdisciplinary teamwork, communication skills, data analysis and critical appraisal, and informed consent through being volunteers in research program - The primary motivators for students were to contribute to COVID-19, to make a difference during the crisis, and to gain clinical research experience - Some difficulties reported by the participants of the research program, such as lack of transportation, conflicts with other obligations or university studies, moving home or away from the site, risk of exposure to COVID-19, and lack of role guidance and teaching |
| Michno et al. (2021)^33^ | Global (74 countries, 260 universities) | Cross-sectional | 760 medical students | Examine the perceptions of medical students on the changes in their medical education and their participation in the pandemic | Online survey questionnaire | - 71.18% of medical students would help the medical workforce during a pandemic - Clinical students were more likely than preclinical students to assist in a medical position (p < 0.05) - Those worried about their wellbeing were less likely to undertake clinical practice |
| Mihatsch et al. (2021)^40^ | Germany | Cross-sectional | 1,407 medical students | Assess medical students’ attitudes toward volunteering and their fear of potential virus infections during a pandemic | Online survey tool | - 67.9% of participants said that they had volunteered - Students who volunteered showed a considerably lower anxiety index than non-volunteering students. The level of anxiousness was higher in females (p < 0.001) - The proportion of male students who volunteered (75.0%) was higher than females (65.7%) - Both male and female students worked predominantly in a clinical context (62.5%) |
| Mousa et al. (2021)^24^ | Saudi Arabia | Cross-sectional | 385 health sector students | Determine the benefits and obstacles of volunteering at COVID-19 vaccine centers in Al-Ahsa | Online survey questionnaire | - Students who volunteered were more likely to be female, third-year students, and have a family member who has previously volunteered - Students learned about volunteer opportunities from social media, friends, and volunteering portals - Volunteering offered additional benefits, including the experiences, self-development, other benefits (social purpose), builds and develops CVs, improves communication skills and time management - Study commitments, distance, transportation, parents’ objections, environmental issues, and long volunteering hours were some obstacles for health professional students from being volunteers - Nursing students were the greatest contributor to the volunteering population |
| Mühlbauer et al. (2021)^51^ | Germany | Cross-sectional | 262 medical students in the clinical phase | - Examine what motivates medical students to volunteer in hospitals - Investigate the effect of resilience and depressive symptoms in the pandemic’s impact on volunteerism and non-volunteerism | Online survey questionnaire | - Medical students volunteered because they were motivated by altruistic impulses and a passion for helping others - Introjected motivation was most prevalent in the volunteering group, while commitments to other activities and time overlaps were the most important reasons for not volunteering - No significant differences in COVID-19 resilience and anxiety were observed between the two groups of students - Non-volunteers were more significantly depressed than volunteers (p=0.04) |
| Nikendei et al. (2021)^55^ | Germany | Qualitative | 194 medical students | Examine medical students' expectations, experiences, and  mental burden related to volunteering in COVID-19 patient support and  treatment services | Semi-structured interviews | - The participants were uncomfortable and uncertain before starting their volunteer jobs - Volunteering was initially unstructured, but their duties became apparent over time - Participants reported strong team cohesion, which helped alleviate initial anxieties and doubts. Working in the field also helped participants preserve their professional identity while standard medical classes and bedside learning were interrupted due to COVID-19 problems - The volunteers felt little psychological burden |
| Patel et al. (2020)^52^ | United Kingdom | Qualitative | 132 medical students | Establish the reasons that medical students  volunteered in OneTrust and understand to their concerns | Online survey questionnaire | - Medical students volunteered for broadly four motivations: to contribute, to learn, to benefit from remuneration, and to fill their spare time during the national lockdown - The most common concerns were lack of PPE, ambiguity about expectations, and the possibility of getting an infection |
| Salem et al. (2021)^35^ | Egypt | Cross-sectional | 3,263 medical students | Assess knowledge, attitudes, and preventive actions related to COVID-19, including the volunteering activity | Online survey questionnaire | - Good knowledge and male gender were associated with a higher willingness to volunteer |
| Seah et al. (2021)^31^ | Singapore | Qualitative | 33 nursing students | Identify parameters impacting final-year nursing students' propensity to volunteer during the COVID-19 pandemic and strategies to improve future volunteering uptake and processes | Semi-structured interview guide, focus-group discussions | - Volunteering motivations: to serve, care about migrant workers, apply knowledge and learn skills, seek experiences - Volunteering concerns: pandemic uncertainty, confidence in the healthcare system, peer pressure, academic concerns, incentives, and accountability to scholarship/sponsorship sources |
| Shi et al. (2021)^42^ | China | Cross-sectional | 2,454 medical students | Examine the inﬂuence of prosocial motivation of medical students on their volunteer behavior and verify the chain mediating role of calling and vocation as well as social responsibility in the relationship  between prosocial motivation and voluntary behavior | Online survey questionnaire | - Medical students who have a strong prosocial motive are more inclined to volunteer during public health crises - Calling, vocation, and social responsibility play a chain-mediating role in the influence of prosocial motivation on volunteer behavior - Medical schools, government, and nonprofit groups must work together to encourage volunteerism among medical students - All entities should work together to create extensive job opportunities and platforms for medical students to generate volunteer services, as well as to build a sustainable incentive system to encourage medical students to engage in volunteer behavior to serve the society |
| Tempski et al. (2021)^14^ | Brazil | Cross-sectional | 10,433 medical students | Evaluate medical students' motivation to join health-care teams to face the COVID-19 pandemic | Online survey questionnaire | - Among students who believed they should work during the pandemic, the most critical factor was the desire to serve (moral principles) - A stronger sense of duty and altruism led students to participate in health-related activities during the pandemic - Emotional factors, fear of contamination, and thoughts that all educational activities should be halted were predictors of students’ refusal to volunteer |
| Tosun et al. (2020)^32^ | Global (64 countries, 4 continents) | Cross-sectional | 1,454 medical students | Evaluate knowledge, behaviors and opinions that have been put in place in response to the COVID-19  pandemic by medical students throughout the world | Online survey questionnaire | - Medical students in Asia (91.5%) seem more likely to be involved in voluntary activity in hospitals. The proportion decreased from Europe, Africa, South America, to Turkey (71.1%), with an overall willingness of 75.1% - African students had the highest proportion (58.6%) of the feeling of being qualified to volunteer in hospitals (overall: 25.7%). Thus, they also had the highest rate (85.7%) of supporting the views that medical students should be qualified to volunteer (overall: 75.9%) |
| Tran et al. (2021)^25^ | Vietnam | Cross-sectional | 2,032 healthcare students | Determine the willingness of healthcare students to volunteer during the COVID-19 pandemic | Online survey questionnaire | - The majority of respondents (72.5%) wanted to volunteer during the COVID-19 pandemic. However, 81.3% of those polled to not participate in COVID-19 preventive and control efforts. Also, less than 50% of individuals were willing to volunteer with patients - Most students volunteered in non-healthcare sectors (65.7% vs. 37% in healthcare) - Attendance at COVID-19 preventive and control training courses, past volunteering experience in non-healthcare industries, year of school, study field, educational format, living arrangements, health status self-perception, and chronic illness possession were associated with willingness to volunteer - Fear for their family’s health and lack of training/knowledge were the main hurdles to volunteering - Many participants stated that they would volunteer if properly trained (79.04%), healthy (78.59%), and equipped with adequate PPE (81.40%) |
| Yu, et al. (2020)^43^ | China | Cross-sectional | 552 medical students | To study medical students interest in the relevant knowledge on COVID-19 and what roles they want to play in the pandemic | Online survey questionnaire | - 85.6% of respondents were willing to volunteer with COVID-19 - Students exhibited an interest in direct patient care (50.2%), indirect medical activities (69.4%), and administrative jobs (80.4%) - Females and those who believed that healthcare professionals should volunteer in a pandemic were substantially more interested in volunteering |
| Zhang et al. (2021)^44^ | China | Cross-sectional | 1,041 undergraduate and graduate medical students | Conduct an assessment of the psychological burden and experience on medical student volunteers during the COVID-19 pandemic | Online questionnaire, semi-structured interviews, in-depth interviews | - Female medical students were more likely to be involved in pandemic prevention and control (52.4% vs. 47.6%) - Depression, anxiety, and stress were detected in 26.8%, 20.2%, and 11.1% of medical student volunteers, respectively - Because of their sense of responsibility as medical students, the vast majority of medical students were willing to volunteer - Student volunteers took others’ temperatures, educated on pandemic prevention and control, gathered data on suspected cases, disinfected and sterilized the pandemic area, collected nucleic acids, assisted medical staff in patient assessments, conducted epidemiological surveys, and collected residents’ health information |
